# Establishing Molecular Subgroups of CD8+ T Cell-Associated Genes in the Ovarian Cancer Tumour Microenvironment and Predicting the Immunotherapy Response

**DOI:** 10.3390/biomedicines11092399

**Published:** 2023-08-28

**Authors:** Yunshu Zhu, Leilei Liang, Jian Li, Jia Zeng, Hongwen Yao, Lingying Wu

**Affiliations:** Department of Gynecologic Oncology, National Cancer Center/National Clinical Research Center for Cancer/Cancer Hospital, Chinese Academy of Medical Sciences and Peking Union Medical College, Beijing 100021, China

**Keywords:** ovarian cancer, immunotherapy, biomarkers, tumour microenvironment, risk model

## Abstract

Background: The mechanism by which infiltrating CD8+ T lymphocytes in the tumour microenvironment influence the survival of patients with ovarian cancer (OC) remains unclear. Methods: To identify biomarkers to optimise OC treatment, 13 immune-cell-line-associated datasets, RNA sequencing data, and clinical data from the GEO, TCGA, and the ICGC were collected. Gene expression in OC was assessed using quantitative reverse transcription polymerase chain reaction (qRT-PCR) and immunohistochemistry (IHC) staining. Results: We identified 520 genes and three immunological clusters (IC1, IC2, and IC3) associated with CD8+ T cells. Higher IFN scores, immune T cell lytic activity, and immune cell infiltration and upregulated expression of immune-checkpoint-related genes indicated that IC3 is more responsive to immunotherapy, whereas IC1 and IC2 have a poorer prognosis. A 10-gene signature, including *SEMA4F*, *CX3CR1*, *STX7*, *PASK*, *AKIRIN2*, *HEMGN*, *GBP5*, *NSG1*, and *CXorf65*, was constructed, and a multivariate Cox regression analysis revealed a significant association between the 10-gene signature-based risk model and overall survival (*p* < 0.001). A nomogram was constructed with age and the 10-gene signature. Consistent with the bioinformatics analysis, IHC and qRT-PCR confirmed the accuracy of the signatures in OC tissue samples. The predictive ability of the risk model was demonstrated using the Imvigor210 immunotherapy dataset. Conclusions: The development of a novel gene signature associated with CD8+ T cells could facilitate more accurate prognostics and prediction of the immunotherapeutic response of patients with OC.

## 1. Introduction

Worldwide, approximately 230,000 new cases of ovarian cancer (OC) are diagnosed and 150,000 patients die every year [1]. Among gynaecological cancers, OC has the greatest mortality rate and only a 46% 5-year survival rate [2], with most patients eventually relapsing and developing resistance to chemotherapy. This loss in efficacy of conventional therapy necessitates the development of novel treatment plans for patients with OC.

Although a high proportion of homologous repair deficiency tumours in OC exhibit a high tumour mutational burden (TMB), increased CD8+ lymphocyte infiltration, and high tumour antigen expression that can independently trigger an antitumour response [3,4,5], clinical studies of OC immunotherapy have not yielded satisfactory results. Additional and more effective biomarkers are therefore required to identify patients that will be sensitive to OC checkpoint inhibitors.

An imbalanced immunological tumour microenvironment (TME) [6], which comprises tumour, stromal, and immune cells [7], is a prominent characteristic of tumours. In most immunotherapeutic settings, CD8+ T lymphocytes eradicate OC cells and are correlated with patient survival [8]. Furthermore, OC is considered a “cold tumour” with a low TMB phenotype [9,10], and can elicit a spontaneous antitumour immune response [5,11]. Human OC tumour antigen-specific CD8+ T lymphocytes that co-express programmed death 1 (PD-1) and LAG-3 produce less interferon-gamma (IFNγ) and TNFα than single-positive cells [12]. However, the mechanism and therapeutic significance of CD8+ T cell infiltration in OC remain unclear.

This study aimed to develop a polygenic signature and predictive model for OC by identifying genes associated with CD8+ T cells using data from immune cell lines. This will allow identification of high-risk subpopulations most likely to benefit from immunotherapy, which will improve prognostics and prediction of immunotherapy efficacy in patients with OC.

## 2. Materials and Methods

### 2.1. Data Pre-Processing

The National Cancer Institute Genomic Data Commons API was used to download TCGA–OV RNA sequencing (RNA-seq) and clinical data. The International Cancer Genome Consortium provided the OV–AU dataset, and the Gene Expression Omnibus (GEO) provided the GSE26193, GSE30161, GSE63885, and GSE9891 datasets for GSE–OV cohort microarray and survival data.

In total, 13 immune-cell-line-related datasets, namely, GSE37750, GSE13906, GSE59237, GSE23371, GSE27838, GSE28726, GSE39889, GSE42058, GSE28490, GSE49910, GSE6863, GSE27291, and GSE8059, were downloaded from the GEO.

The TMB was estimated from the TCGA mutect2-processed mutation dataset, and the single-sample GSEA approach was used to calculate IFN scores from Th1/IFN gene signatures as previously described [13].

Affy’s RMA was used to process each immune-cell-line-associated dataset, and the limma “RemoveBatchEffect” R-package was used to eliminate batch effects.

TCGA–OV and OV–AU RNA-seq data samples lacking follow-up, survival time, or living status were eliminated. Ensemble gene IDs were transformed into gene symbols, and median values were determined when numerous gene symbols were expressed. Batch effect adjustments were made.

The GSE–OV dataset excluded normal tissue samples, samples without follow-up, OS, and living status. An annotation file transformed probes into gene symbols. The Affy RMA algorithm processed each immune-cell-line-associated dataset. Batch effects were corrected.

After quality control, the dataset included 373 TCGA–OV samples, 93 OV–AU samples, and 511 GSE–OV samples (Supplementary Table S1).

A single dataset was created from 13 immune-cell-line-associated datasets by merging the RNA-seq data of TCGA–OV and OV–AU dataset samples into the RNA-seq dataset, and merging the GSE26193, GSE30161, GSE63885, and GSE9891 microarray data into a single GSE–OV dataset. Principal component analysis verified the batch effect correction of the three merged datasets. The datasets were dispersed (Supplementary Figure S1A,C,E) but harmonised after batch effect correction (Supplementary Figure S1B,D,F).

### 2.2. Weighted Gene Correlation Network Analysis (WGCNA)

The WGCNA R-package was used to construct a weighted co-expression network based on the expression patterns of co-expressed coding genes and modules. Co-expression module screening used an eight-threshold soft threshold. The co-expression network was scale-free, with the log(k) of the node, where K represents the connection, negatively associated with log (P(k)), where P represents probability of occurrence, with a correlation coefficient of >0.8. The expression matrix was converted into an adjacency matrix and subsequently a topology matrix. Using average linkage hierarchical clustering, we constructed a topological overlap matrix of clustered genes. Hybrid dynamic tree cut-off criteria included 200 genes per gene network module. The dynamicTreecut R-package was used to identify gene modules and calculate the eigenvector values of each module. Modules were clustered and merged by applying height = 0.25, deepSplit = 2, and minModuleSize = 200.

### 2.3. Immunotyping

We performed a univariate Cox analysis of CD8+ T-cell genes and intersecting genes from the RNA-seq and GSE–OV datasets. The ConsensusClusterPlus R-package grouped the 466 RNA-seq samples according to the predicted CD8+ T-cell gene expression. The cumulative distribution function (CDF) was used to determine the optimal cluster number and the CDF delta area curve was used to determine the most stable number of groups. Subsequently, we evaluated the immune subtype characteristics of each group. The same analyses were performed for the GSE–OV group. The pooled Akaike information criterion (AIC) was used in stepwise regression using the MASS package. Briefly, the stepAIC technique involves starting with the model with the most variables and sequentially deleting variables to decrease the AIC, as a smaller AIC is indicative of a better fitted model. The workflow is shown in Supplementary Figure S2.

### 2.4. Specimen Collection

Surgical OC resection specimens and adjacent normal tissues were snap-frozen in liquid nitrogen and kept at −80 °C until RNA extraction. This study was approved by the Ethics Committee of the Cancer Hospital, Chinese Academy of Medical Sciences and Peking Union Medical College (17-099/1355).

### 2.5. Quantitative Reverse Transcription PCR (qRT-PCR) and Immunohistochemistry Analysis

Total RNA from 10 ovarian tumours and 10 non-tumour tissues was isolated using RNA Easy Isolation Reagent (Vazyme, Nanjing, China). qRT-PCR was performed using Vazyme’s HiScript III 1st Strand cDNA Synthesis Kit and ChamQTM Universal SYBR qPCR Master Mix. Primer sequences are listed in Supplementary Table S2. GAPDH was used as the internal control. Immunohistochemistry (IHC) was conducted as previously described [14]. Anti-TXK (1:500), anti-STX7 (1:500), and anti-HEMGN (1:500) antibodies were purchased from Proteintech, and anti-SEMA4F (1:500) antibodies were purchased from Abcam. Images were acquired at 20× magnification.

### 2.6. Statistical Analysis

Statistical analyses were performed with R software 3.5.3 and GraphPad Prism v. 8.01 (GraphPad Software, La Jolla, CA, USA). A Student’s *t* test was used to compare values between test and control groups. *p* values of <0.05 indicated statistical significance.

## 3. Results

### 3.1. CD8+ T Cell-Associated Genes

Hierarchical clustering generated 179 immune-cell-line-associated expression profiles (Supplementary Figure S3A). To obtain a scale-free network, we set β = 8 (Supplementary Figure S3B); 12 modules were obtained (Supplementary Figure S3C). Grey module genes could not be aggregated into other modules. The pink module contained 520 genes, most of which were positively linked to CD8+ T cells (Supplementary Figure S3D).

Gene ontology (GO) functional annotation determined the top ten biological processes (BPs, Supplementary Figure S4A), cellular components (CCs, Supplementary Figure S4B), and molecular functions (MFs, Supplementary Figure S4C) enriched in the CD8+ T cell module. Additionally, a Kyoto Encyclopedia of Genes and Genomes (KEGG) enrichment analysis (Supplementary Figure S4D) identified 10 significantly enriched immunological pathways involving primary immunodeficiency and T cell receptor signalling.

### 3.2. Immunophenotyping

Univariate analyses of the RNA-seq and GSE–OV datasets revealed 71 and 84 prognostic genes, respectively. Nine genes were included in both datasets (Supplementary Figure S5A), indicating that CD8+ T cell-associated gene expression may vary across sequencing platforms. We also analysed 146 prognostically important genes from both datasets (*p* < 0.05).

When k = 3, the RNA-seq dataset consensus clustering analysis yielded three stable immunological clusters (ICs; Supplementary Figure S5B,C). The three ICs exhibit prognostic ability (Supplementary Figure S5D), with IC3 exhibiting a better prognosis than IC1 and IC2. These findings were successfully replicated in an independent GSE–OV cohort using the same methodology (Supplementary Figure S5E).

### 3.3. Relationship between Immunophenotyping and TMB and Gene Mutation

**Figure 1:**
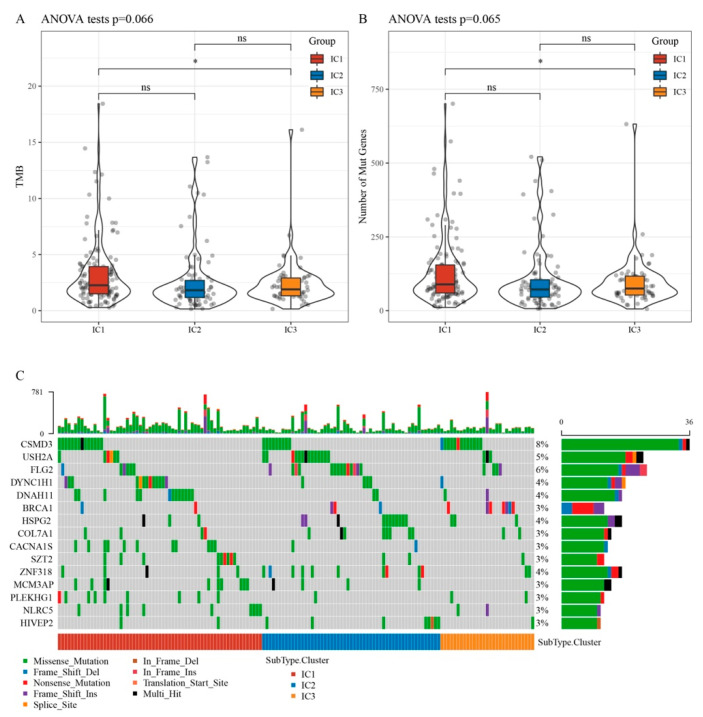
Relationship between TMB and molecular subtypes. (**A**) Distribution of TMB for subtype samples. (**B**) Distribution of the number of mutations for subtype samples. (**C**) Mutation features of significantly mutated genes in samples of each subtype. * *p* < 0.05. ns: no significance.

The TMB differed significantly between IC1 and IC3 (Figure 1A). Comparison of the mutated gene counts indicated that IC1 had significantly more mutated genes than IC3 (Figure 1B). Chi-square tests of 2431 genes with mutation frequencies of >3 and significantly high-frequency mutations in each IC yielded 202 genes (*p* < 0.05). The mutation signatures of the 15 genes in each IC group are shown in Figure 1C.

### 3.4. Immunophenotyping Chemokines and Immunological Checkpoint Genes

**Figure 2:**
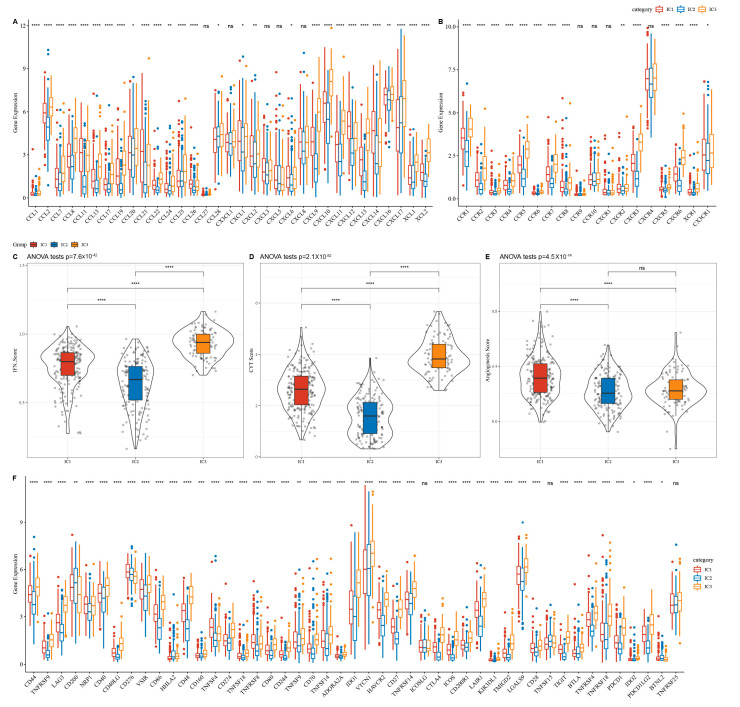
Differences in immune molecule expression and function between molecular subtypes in the RNA-seq cohort for (**A**) chemokines, (**B**) chemokine receptors, (**C**) IFNγ, (**D**) immune T-cell lysis activity, (**E**) angiogenesis scores, and (**F**) immune checkpoint genes. Significance was determined using an ANOVA, with * *p* < 0.05; ** *p* < 0.01, *** *p* < 0.001, and **** *p* < 0.0001. ns: no significance.

Examination of the RNA-seq cohort revealed differential chemokine expression for 28 of 33 chemokines (84.85%; Figure 2A), suggesting differences in immune cell infiltration and immunotherapeutic responses in the three ICs. Immunophenotyping also identified differential expression for 14 of 18 chemokine receptor genes (Figure 2B).

CD8+ T lymphocytes may release IFNγ to upregulate PD-1/PD-L1 [15,16] and IDO1 [17] gene expression, with IDO1 overexpression linked to poor prognosis, tumour development, and metastasis. The Th1/IFNγ gene signatures were extracted as previously described [13], and IFNγ scores were calculated for each sample using the single-sample GSEA method. We found significant differences in IFNγ scores among ICs; the IC3 group exhibited higher IFNγ scores than the IC1 and IC2 groups (Figure 2C).

Evaluation of mean *GZMA* and *PRF1* expression levels, used to assess intratumoural immune T-cell lysis activity [18], revealed substantial variation among the three ICs (Figure 2D), with the IC3 group exhibiting greater immunological T-cell lysis activity than the IC1 and IC2 groups.

Based on the angiogenesis-related gene set obtained from a previous study [19], the angiogenesis score of each patient was calculated by the ssGSEA method in the R-package GSVA, and significant differences were found between the subgroups. It was found that the angiogenesis scores of IC2 and IC3 subgroups were lower than those of the IC1 subgroup (Figure 2E).

Examination of 47 immune-checkpoint-related genes in different ICs in a prior study [13] revealed significant differences for 44 (93.62%) patients (Figure 2F), with different subgroups exhibiting differential responses to immunotherapy. Immune-checkpoint-related genes, including *CTLA4*, *PDCD1*, *PDCD1LG2*, and *IDO1*, were highly expressed in IC3.

### 3.5. Immune Signatures and Pathways in Different ICs

**Figure 3:**
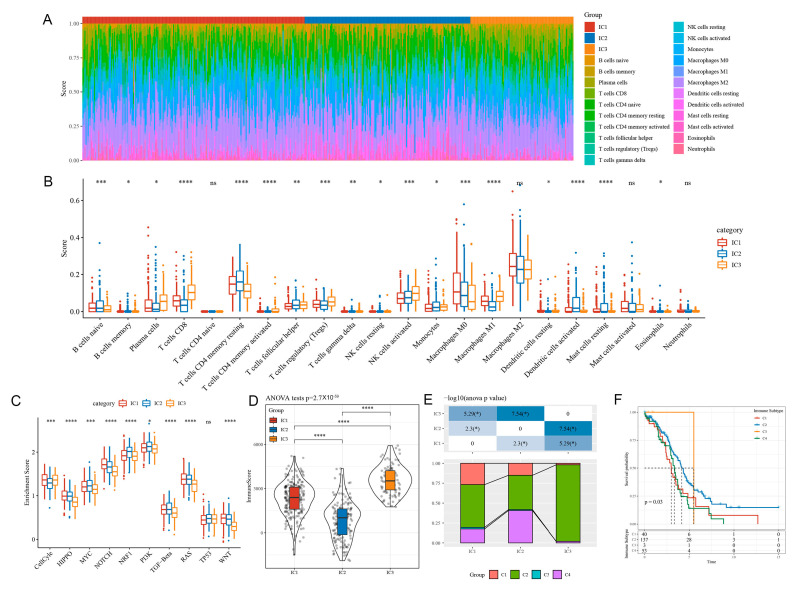
Immunological features and pathway characteristics of molecular subtypes. (**A**) Proportions of 22 immune cell types in subtype samples. (**B**) Differences in immune cell scores of 22 immune cell components between subtype samples. (**C**) Differences in enrichment scores of ten pathways associated with tumour abnormalities between subtypes. (**D**) Distribution of immune infiltration scores between subtype samples. (**E**,**F**) Comparison of the molecular subtypes with six previously identified pan-cancer immunophenotypes. * *p* < 0.05; ** *p* < 0.01, *** *p* < 0.001, and **** *p* < 0.0001. ns: no significance.

A CIBERSORT analysis of 22 immune cell scores in each sample of the RNA-seq dataset revealed substantial immunological signature differences between the subgroups (Figure 3A). CD8+ T cells, resting memory CD4+ T cells, and M0, M1, and M2 macrophages were all highly expressed (Figure 3B).

Analysis of 10 carcinogenic pathways reported in a previous study [20] showed that 9 pathways were significantly different between the three subtypes (Figure 3C). IC3 exhibited the highest microenvironment infiltration (Figure 3D).

A comparison of six previously reported immunophenotypes of pan-carcinoma [21] and the ICs in the current study indicated that the ICs intersected with four of the six published immune subtypes (Figure 3E,F). This suggests that the three ICs in this study are complimentary to the four previously published immunophenotypes.

### 3.6. Tumour Immune Dysfunction and Exclusion (TIDE) among ICs

**Figure 4:**
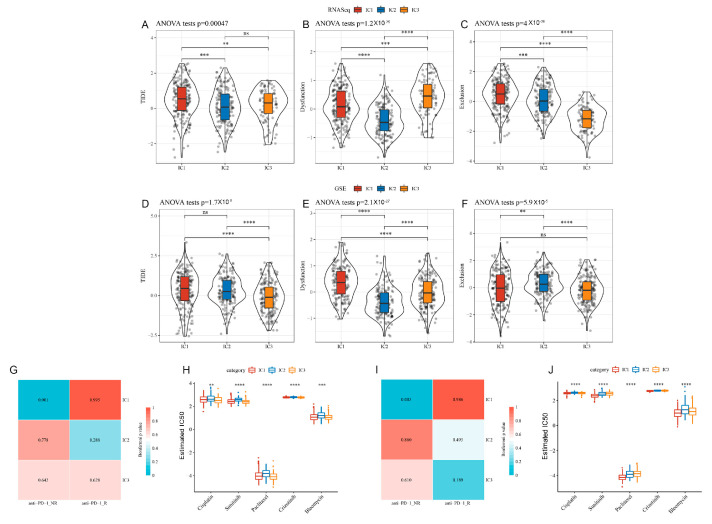
(**A**) TIDE scores in the RNA-seq dataset samples. (**B**) T cell dysfunction scores in the RNA-seq dataset samples. (**C**) T cell rejection scores in the RNA-seq dataset samples. (**D**) TIDE scores in the GSE-OV dataset samples. (**E**) T cell dysfunction scores in the GSE-OV dataset samples. (**F**) T cell rejection scores in the GSE-OV dataset samples. (**G**) RNA-seq submap analysis showing that IC1 may be insensitive to anti-PD-1 (Bonferroni corrected, *p* < 0.05). (**H**) The response of different immune clusters in the RNA-seq dataset to traditional chemotherapy drugs. (**I**) GSE submap analysis showing that IC1 may be insensitive to PD-1 inhibitors (Bonferroni corrected, *p* < 0.05). (**J**) The response of different immune clusters in the GSE-OV dataset to traditional chemotherapy drugs. ** *p* < 0.01, *** *p* < 0.001, and **** *p* < 0.0001. ns: no significance.

The clinical effects of immunotherapy on the three ICs were assessed using TIDE (http://tide.dfci.harvard.edu/ (accessed on 20 August 2021)), with a higher TIDE prediction score indicating a greater likelihood of immune escape and lower immunotherapy efficacy. Analysis of the RNA-seq dataset indicated that IC2 and IC3 could benefit more from immunotherapy than IC1 (Figure 4A). Comparison of projected T cell dysfunction and rejection ratings revealed that IC2 exhibits reduced T cell dysfunction, and the projected T cell rejection scores were greater for IC1 and IC2 than for IC3 (Figure 4B,C). Consistent trends were observed for the GSE dataset (Figure 4D–F).

### 3.7. Differential Analysis of ICs in Immunotherapy/Chemotherapy

We analysed variations in immunotherapy and chemotherapy efficacy. The GSE78220 dataset was used to examine immunological cluster similarity and immunotherapy efficacy via subclass mapping, with increasing similarity reflected by decreasing p-values. IC1 was similar to anti-PD-1_no response (anti-PD-1_NR) in both the RNA-seq (Figure 4G) and GSE (Figure 4I) datasets, suggesting it may be insensitive to PD-1 inhibitors. Evaluation of cisplatin, sunitinib, paclitaxel, crizotinib, and bleomycin indicated that IC2 was less responsive to these five medications than IC1 and IC3 (Figure 4H,J).

### 3.8. Prognostic Risk Model 

Training and validation sets were constructed from the 466 samples in the RNA-seq dataset. All samples were randomly clustered 100 times with replacement to prevent random allocation bias from affecting modelling stability. The training-to-validation ratio was 4:1 for group sampling.

Using the coxph function of the survival R-package and a filtering threshold of *p* < 0.01 on the training set, a univariate Cox proportional hazards regression model was constructed for each CD8+ T cell-related gene and survival data. In total, 11 prognostic genes were identified in the RNA-seq and GSE datasets. A multivariate Cox analysis (Supplementary Figure S6) and the stepwise regression algorithm reduced the 11 genes to 10 genes, including *TXK*, *STX7*, *PASK*, *AKIRIN2*, *SEMA4F*, *HEMGN*, *GBP5*, *CX3CR1*, *NSG1*, and *CXorf65*.

The 10-gene signature formula was as follows:

RiskScore = −0.232 × TXK + 0.354 × STX7 − 0.224 × PASK − 0.279 × AKIRIN2 + 0.29 × SEMA4F − 6.158 × HEMGN − 0.17 × GBP5 + 0.183 × CX3CR1 − 0.084 × NSG1 − 0.342 × CXorf65

The risk score distribution (Supplementary Figure S7A) was generated for each sample based on its expression level in the RNA-seq training dataset. The timeROC R-package was used to classify RiskScore prognosis, and the prognostic prediction of the risk model at 1, 3, and 5 years assessed using a receiver operating characteristic (ROC) curve analysis (Supplementary Figure S7B) revealed a high area under the curve (AUC) for the model. RiskScore z-scores classified samples as high risk (>0) or low risk (<0), and subsequently constructed Kaplan–Meier (KM) curves indicated that the high-risk group had a significantly reduced survival rate (*p* < 0.0001) (Supplementary Figure S7C).

### 3.9. Prognostic Risk Model Validation

The RiskScore distribution of samples in the RNA-seq validation set is shown in Supplementary Figure S8A. An ROC analysis showed high AUC values for the risk model at 1, 3, and 5 years (Supplementary Figure S8B). KM curves showed lower survival in the high-risk group (*p* < 0.01; Supplementary Figure S8C).

Supplementary Figure S9A shows the RiskScore distribution of all the RNA-seq datasets. An ROC analysis revealed a high AUC for the risk model at 1, 3, and 5 years (Supplementary Figure S9B), and the KM curves indicated significantly reduced survival rates for the high-risk group (*p* < 0.0001; Supplementary Figure S9C).

Supplementary Figure S10A shows the RiskScore distribution of samples in the independent GSE validation dataset. The RiskScore prognostic classification was evaluated using an ROC analysis (Supplementary Figure S10B), which indicated a high AUC value. The KM analysis results are consistent with the results of the RNA-seq datasets (Supplementary Figure S10C, *p* < 0.001).

In addition, KM and ROC curves were constructed for RiskType using the survival information of TCGA cohort (Supplementary Figure S11A), ICGC cohort (Supplementary Figure S11B), and four GSE cohorts (GSE26193, GSE30161, GSE63885, and GSE9891) (Supplementary Figure S11C–F).

### 3.10. Relationships between RiskScore, Clinical Characteristics, and Molecular Subtypes

Comparison of the RiskScore distribution of all TCGA datasets among clinical features indicated higher risk scores in later clinical stages (Supplementary Figure S12B). IC3 had the best prognosis and lowest risk score (Supplementary Figure S12C), whereas IC1 had the poorest prognosis. Other clinical features did not differ significantly (Supplementary Figure S12A,D). For the TCGA dataset, we carried out a KM survival analysis stratified by age, stage, and grade. Patients were stratified into ≤60 or >60 years subgroups (Supplementary Figure S12E,F), stage I–II and III–IV subgroups (Supplementary Figure S12G,H), and G1/2 and G3/4 subgroups (Supplementary Figure S12I,J). High-risk patients aged 60 years or older, stage III–IV, and G1/2 and G3/4 subgroups exhibited a shorter OS than low-risk patients. Our risk model exhibited good predictive ability in diverse clinical clusters.

In the 10-gene signature-based risk model, age and RiskScore were significantly correlated with OS in univariate (Supplementary Figure S13A) and multivariate (Supplementary Figure S13B; HR = 1.88, 95% CI = 1.43–2.48, *p* < 0.001) Cox regression analyses, indicating that our 10-gene signature approach is clinically predictive.

### 3.11. Nomograms and Forest Plots

**Figure 5:**
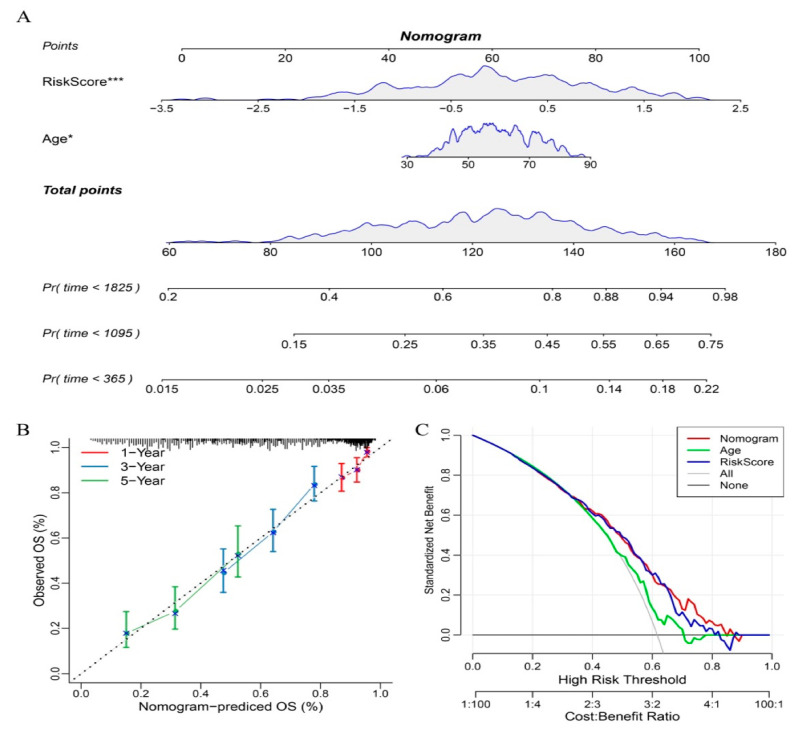
Nomogram and forest plot constructed with the RiskScore and clinical features using the TCGA dataset. (**A**) Nomogram. (**B**) Calibration curves showing the observed OS versus predicted probability of 1-, 3-, and 5-year survival of the nomogram. (**C**) Decision curve analysis plot. * *p* < 0.05, *** *p* < 0.001.

Using the full TCGA dataset, we constructed an age–RiskScore nomogram (Figure 5A). The RiskScore function of the 10-gene-based risk model had the greatest survival prediction ability. Calibration curves and DCA graphs are shown in Figure 5B,C. The nomogram performed well.

### 3.12. Predicting Immunotherapy Efficacy with the Constructed Risk Model

An immunotherapy dataset (Imvigor210) with transcriptome data was obtained to test the 10-gene signature risk model. KM curves showed that immunotherapy patients with higher RiskScores had poorer survival rates (Supplementary Figure S14A). RiskScore ROC curves showed higher AUC values (Supplementary Figure S14B). Immunotherapy responders and non-responders differed significantly in both high-risk and low-risk groups (Supplementary Figure S14C). The MCPcounter R-package calculated immune cell scores for Imvigor210 samples. TMB, NEO, and immune cell scores were inversely related to RiskScore (Supplementary Figure S14D).

Furthermore, among groups, immunotherapy efficacy, immune cell grouping, tumour cell grouping, and immunophenotypic grouping differed significantly according to the RiskScore (Supplementary Figure S15A–D).

### 3.13. Gene Expression in OC Tissues

**Figure 6:**
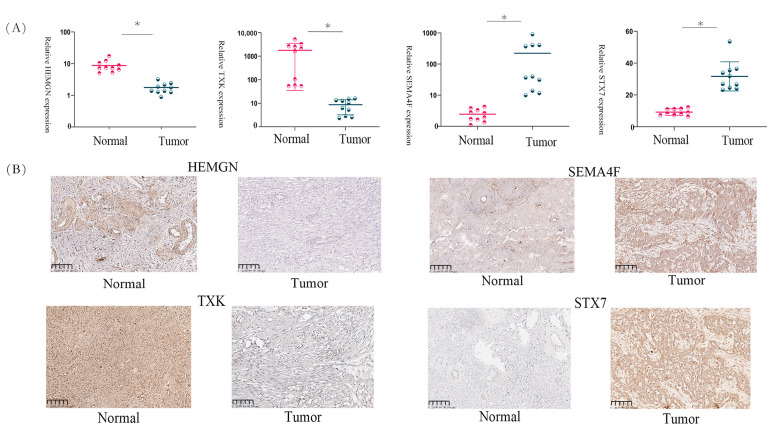
(**A**) qPCR and (**B**) IHC results showing low HEMGN and TXK and high SEMA4F and STX7 expression in OC tissues. * *p* < 0.05.

To validate the 10-gene signature, we analysed the expression of four genes in paired tumour and adjacent non-tumour tissues from patients with OC. The clinical information of the patients with OC is shown in Supplementary Table S3. IHC and qRT-PCR revealed downregulated expression of HEMGN and TXK in OC tissues compared to normal tissues (Figure 6A,B), whereas SEMA4F and STX7 were highly expressed in OC tissues. These results validate the bioinformatics analyses.

## 4. Discussion

Bevacizumab has been one of the most studied targeted agents in OC in recent decades; however, despite its widespread use and efficacy, patient selection and timing of treatment remain controversial and the survival benefit in patients with advanced OC remains limited [22]. Currently, immune checkpoint blockade (ICB) therapy is effective in cancers such as melanoma and non-small cell lung cancer, but the therapeutic value of immunotherapy in OC is still in the research stage. A phase II clinical study evaluating the role of combined anti-PD-1 antibodies and anti-VEGF antibodies in recurrent epithelial OC showed an overall response rate of 28.9%, with a 40% overall response rate in platinum-sensitive OC [23]. The important reason limiting the response to immunotherapy and disease progression in OC is that the tumour microenvironment of OC is in a state of immunosuppression [24]. Immunologically activated tumour-infiltrating lymphocytes (TILs) in ovarian tumour tissue demonstrate that the immune system is the trigger for this tumour [25]. Therefore, the present study was conducted to construct a gene model associated with CD8+ T lymphocytes to identify patients with OC who would benefit from immunotherapy and predict prognosis.

We identified three OC ICs and a risk prediction model based on genes associated with CD8+ T cells. The prognosis was worse for IC1 and IC2 than for IC3. TMB and the number of gene mutations were considerably higher in IC1 than in IC3. Chemokines, their receptor genes, and angiogenesis-related genes varied substantially between clusters. IC3 had a better prognosis than IC1 and IC2 owing to greater IFN scores, immune T cell lytic activity, immune cell infiltration, and immune checkpoint gene expression. IC1 had a higher TIDE score than IC2 and IC3, indicating a larger risk of immune escape and less therapeutic benefits. IC1 and IC3 were more sensitive to cisplatin, sunitinib, paclitaxel, crizotinib, and bleomycin than IC2, suggesting that the three ICs can identify high-risk patients with OC and help clinicians choose treatment drugs.

In total, 10 CD8+ T cell-related genes (*TXK*, *STX7*, *PASK*, *AKIRIN2*, *SEMA4F*, *HEMGN*, *GBP5*, *CX3CR1*, *NSG1*, and *CXorf65*) were identified in patients with OC. High expression of *STX7*, *SEMA4F*, and *CX3CR1* was associated with a high risk and poor prognosis, whereas high expressions of *TXK*, *PASK*, *AKIRIN2*, *HEMGN*, *GBP5*, *NSG1*, and *CXorf65* were associated with a low risk and better prognosis. qRT-PCR and IHC confirmed the accuracy of the signatures in OC tissue samples and the bioinformatics evaluation. Human *TXK* primarily regulates IFNγ gene transcription in Th1/Th0 cells [26]. Syntaxin 7, encoded by *STX7*, affects lysosome trafficking and phagosome–lysosome fusion [27]. *PASK* affects lipid and glucose metabolism, mitochondrial respiration, phosphorylation, and gene expression [28]. *AKIRIN2* controls embryonic development and innate immunity, but its role in carcinogenesis is unclear [29]. *AKIRIN2* expression is correlated with chemotherapy resistance in patients with OC [30]. *SEMA4F* regulates embryologic axon guidance and cancer-induced neurogenesis [31]. *HEMGN* is upregulated in thyroid carcinoma tissues and cells [32] and regulates cellular proliferation and apoptosis via the PI3K/Akt signalling pathway [33]. *GBP5* has prognostic power for OS in OC [34]. The chemokine receptor *CX3CR1* can identify distinct populations within monocyte, macrophage, and dendritic cell lineages [35]. *NSG1* is a neuronal cell-expressed endosomal protein and a direct transcriptional target gene of the tumour suppressor p53 [36].

Although our study was a retrospective investigation, we mitigated potential biases by validating the model with data from multiple cohorts.

## 5. Conclusions

We identified three immunophenotypes of OC and constructed a 10-gene signature-based risk model that accurately predicts the prognosis and response to immunotherapy of patients with OC.

## Data Availability

Most of the datasets used and/or analysed during the current study were obtained from public databases. All data in the current study are available from the corresponding authors upon reasonable request.

## Supplementary Materials

The following supporting information can be downloaded at: https://www.mdpi.com/article/10.3390/biomedicines11092399/s1, Supplementary Figure S1. PCA analysis. (A) PCA before batch effect correction. (B) PCA after batch effect correction. (C) PCA before batch effects on RNA-seq datasets. (D) PCA after batch effects on RNA-seq datasets. (E) PCA before batch effects on the GSE-OV dataset. (F) PCA after batch effects on the GSE-OV dataset. Supplementary Figure S2. Workflow chart of the CD8+ T-cell-related gene model related to OC prognosis. Supplementary Figure S3. WGCNA-based co-expression analysis of CD8+ T-cell-associated genes. (A) Sample clustering analysis. (B) Analysis of network topology for various soft-threshold powers. (C) Gene dendrogram and module colours. (D) Correlation results between the 12 modules and each clinical phenotype. Supplementary Figure S4. Functional enrichment analysis of CD8+ T-cell-associated genes. (A) Biological process annotation map of genes in the pink-coloured module. (B) Molecular function annotation map of genes in the pink-coloured module. (C) Cellular component annotation map of genes in the pink-coloured module. (D) Kyoto Encyclopedia of Genes and Genomes annotation map of genes in the pink-coloured module. Supplementary Figure S5. The ICs in OV. (A) Venn diagram displaying the intersection of CD8+ T-cell genes significantly associated with prognosis between the RNA-Seq and GSE-OV cohorts. (B) CDF curve and CDF delta area curve of the RNA-seq dataset samples. (C) Heatmap of sample clustering at consensus k = 3. (D) Survival curves for the molecular subtypes in the RNA-seq dataset cohort. (E) Survival curves for the molecular subtypes in the GSE-OV dataset cohort. Supplementary Figure S6. Multivariate Cox Forest plot for the 10-gene model. Supplementary Figure S7. Construction and evaluation of the prognostic risk model based on CD8+ T-cell-associated genes using the training set. (A) RiskScore, time to live (TTL), and survival status after applying the 10-gene signature to the RNA-seq training dataset. (B) ROC and AUC based on the 10-gene signature. (C) KM survival curves for high- and low-risk groups based on the 10-gene signature using the RNA-seq training dataset. Supplementary Figure S8. (A) RiskScore, TTL, and survival status after applying the 10-gene signature to the RNA-seq validation dataset. (B) ROC and AUC based on the 10-gene signature. (C) KM survival curves for high- and low-risk groups based on the 10-gene signature using the RNA-seq validation dataset. Supplementary Figure S9. (A) RiskScore, TTL, and survival status after applying the 10-gene signature to all the RNA-seq datasets. (B) ROC and AUC based on the 10-gene signature. (C) KM survival curves for high- and low-risk groups based on the 10-gene signature using all the RNA-seq datasets. Supplementary Figure S10. (A) RiskScore, TTL, and survival status after applying the 10-gene signature to the independent GSE validation dataset. (B) ROC and AUC based on the 10-gene signature. (C) KM survival curves for high- and low-risk groups based on the 10-gene signature using the independent GSE validation dataset. Supplementary Figure S11. KM and ROC curves for RiskScore and the survival of (A) TCGA cohort, (B) ICGC cohort, and (C–F) four GSE cohorts. Supplementary Figure S12. Distribution of the RiskScore of the TCGA dataset among clinical features, including (A) grade, (B) stage, (C) ICs, (D) age. Survival analysis of the risk groupings of the TCGA dataset stratified according to clinical characteristics, including (E) age ≤ 60, (F) age > 60, (G) stage I-II, (H) stage III-IV, (I) grade I-II, and (J) grade III-IV. Supplementary Figure S13. Univariate and multivariate analyses of the risk model based on the 10-gene signature using all TCGA datasets. (A) Univariate Cox regression analysis. (B) Multivariate Cox regression analysis. Supplementary Figure S14. Prediction efficacy of the risk model based on the 10-gene signature. (A) Kaplan–Meier curves of high- and low-risk groups using the Imvigor210 dataset. (B) Evaluation of the risk model compared with standard prediction models of immunotherapy response using the Imvigor210 dataset. (C) Corresponding stacked plots of immunotherapy response among high- and low-risk groups in the Imvigor210 dataset. (D) Correlation between RiskScore, immune score, TMB, and NEO using the Imvigor210 dataset. CR, complete response; PR, partial response; SD, stable disease; PD, progressive disease. Supplementary Figure S15. Comparison of RiskScore distribution across different subgroups for (A) immunotherapy response, (B) immune cell level, (C) tumour cell level, and (D) immune phenotype. Supplementary Table S1. After quality control, the dataset included 373 TCGA–OV samples, 93 OV–AU samples, and 511 GSE–OV samples. Supplementary Table S2. Primer sequences for qRT-PCR analysis. Supplementary Table S3. Clinical information on 10 patients with OC used for immunohistochemistry.

## Abbreviations

Ovarian cancer: OC; AUC: area under the curve; CI: confidence interval; GEO: Gene Expression Omnibus; GO: Gene Oncology; HR: hazard ratio; KEGG: Kyoto Encyclopedia of Genes and Genomes; OS: overall survival; PD-1: programmed death 1; PD-L1: programmed death ligand 1; qRT-PCR: quantitative real-time polymerase chain reaction; ROC: receiver operating characteristic; TCGA: The Cancer Genome Atlas; TIDE: tumour immune dysfunction and exclusion analysis; TMB: tumour mutation burden; TME: tumour microenvironment; NCI GDC API: National Cancer Institute Genomic Data Commons Application Programming Interface; IC1: immune cluster 1; HRD: homologous repair deficiency; TIL: tumour-infiltrating lymphocyte.
