# Supplementary material for: Establishing Molecular Subgroups of CD8+ T Cell-Associated Genes in the Ovarian Cancer Tumour Microenvironment and Predicting the Immunotherapy Response
Source: Biomedicines. 2023 Aug 28;11(9):2399. doi: 10.3390/biomedicines11092399 (PMC10525231; doi:10.3390/biomedicines11092399)

Supplementary Figures legends

Supplementary Figure S1 PCA analysis.

(A) PCA before batch effect correction.

(B) PCA after batch effect correction

(C) PCA before batch effects on RNA-seq datasets.

(D) PCA after batch effects on RNA-seq datasets.

(E) PCA before batch effects on the GSE-OV dataset.

(F) PCA after batch effects on the GSE-OV dataset.

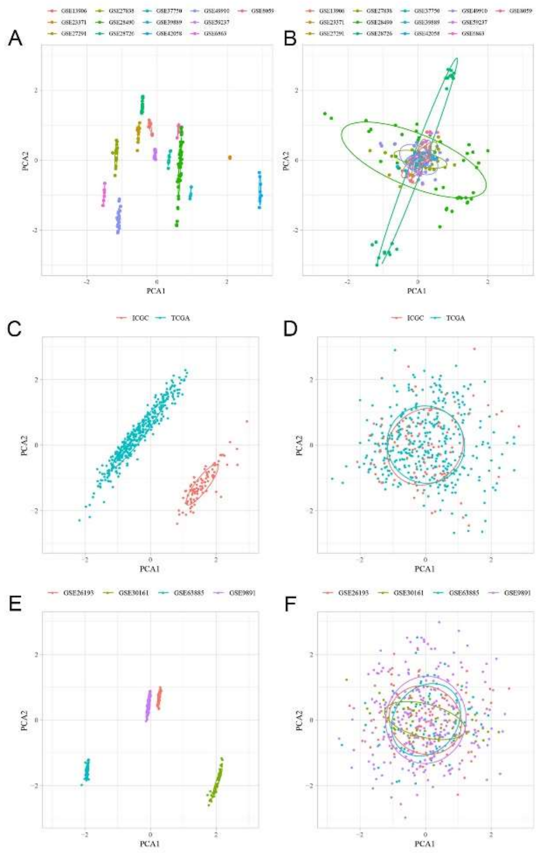

Supplementary Figure S2

Workflow chart of the CD8+ T-cell-related gene model related to OC prognosis.

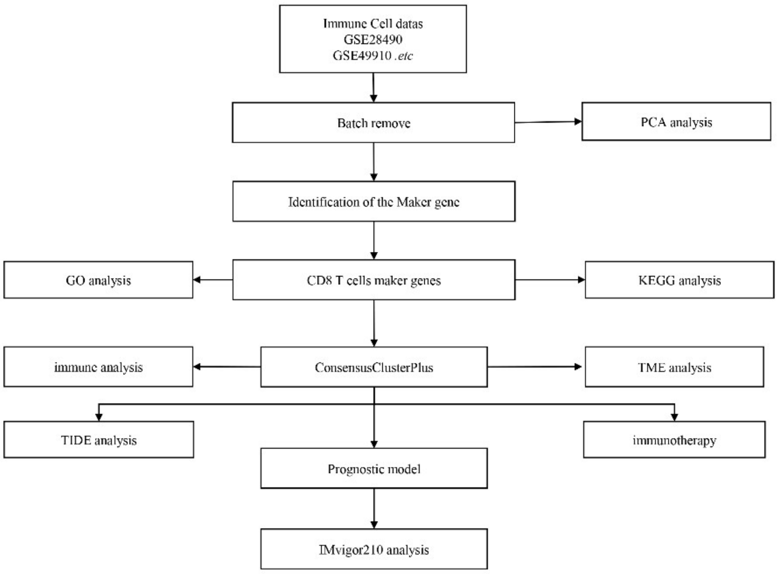

Supplementary Figure S3

WGCNA-based co-expression analysis of CD8+ T-cell-associated genes.

(A) Sample clustering analysis.

(B) Analysis of network topology for various soft-threshold powers.

(C) Gene dendrogram and module colours.

(D) Correlation results between the 12 modules and each clinical phenotype.

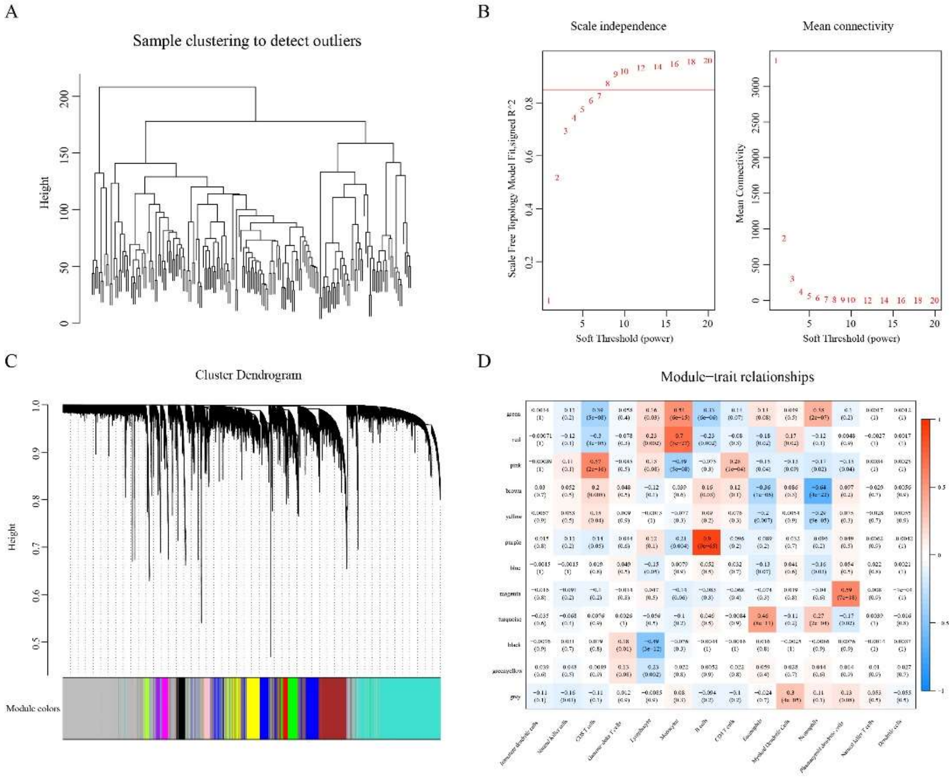

Supplementary Figure S4

Functional enrichment analysis of CD8+ T-cell-associated genes.

(A) Biological process annotation map of genes in the pink-coloured module.

(B) Molecular function annotation map of genes in the pink-coloured module.

(C) Cellular component annotation map of genes in the pink-coloured module.

(D) Kyoto Encyclopedia of Genes and Genomes annotation map of genes in the pink-coloured module.

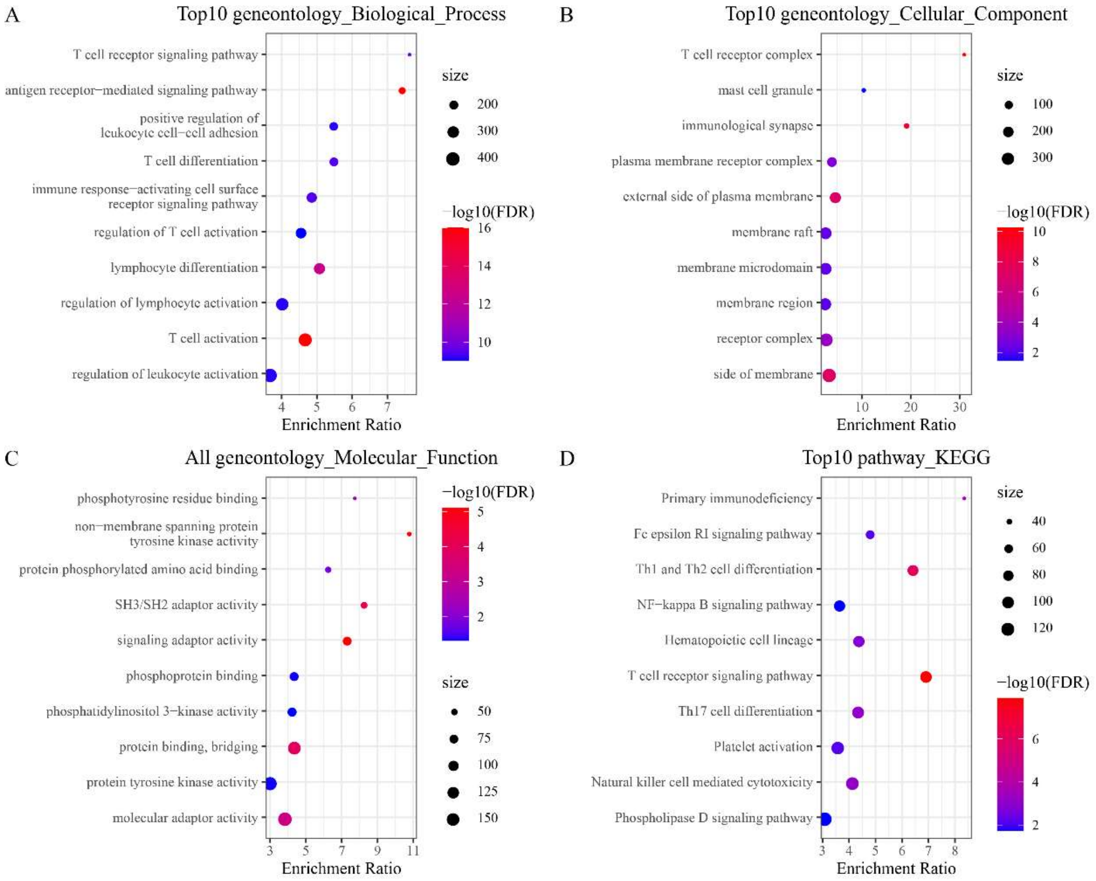

Supplementary Figure S5

The ICs in OV.

(A) Venn diagram displaying the intersection of CD8+ T-cell genes significantly associated with prognosis between the RNA-Seq and GSE-OV cohorts.

(B) CDF curve and CDF delta area curve of the RNA-seq dataset samples.

(C) Heatmap of sample clustering at consensus k = 3.

(D) Survival curves for the molecular subtypes in the RNA-seq dataset cohort.

(E) Survival curves for the molecular subtypes in the GSE-OV dataset cohort.

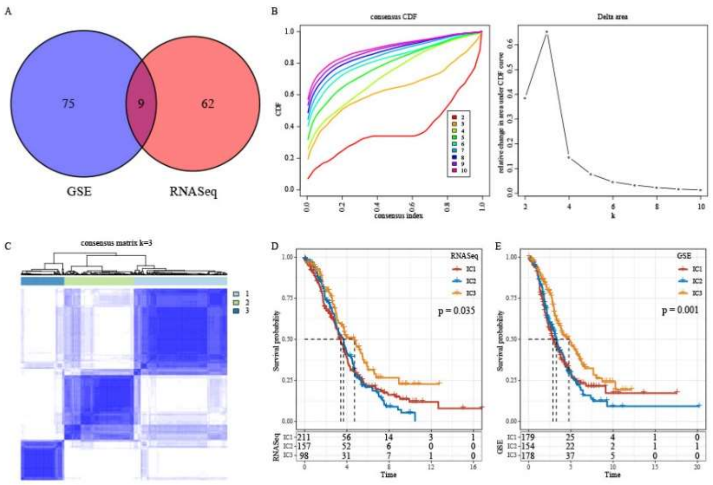

Supplementary Figure S6

Multivariate Cox Forest plot for the 10-gene model.

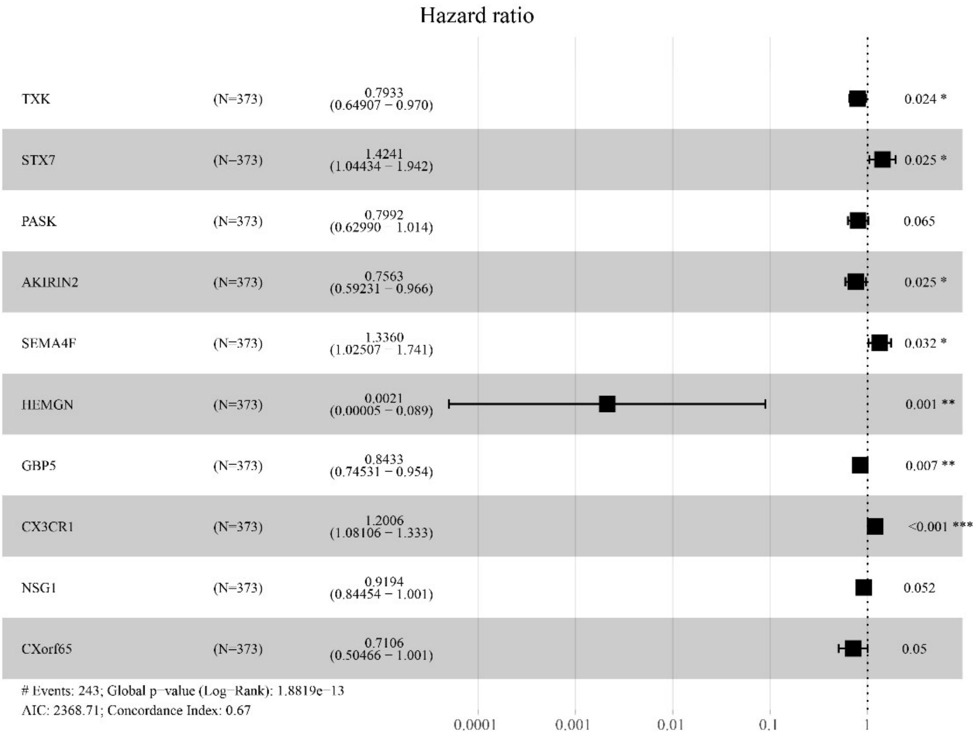

Supplementary Figure S7

Construction and evaluation of the prognostic risk model based on CD8+ T-cell-associated genes using the training set.

(A) RiskScore, time to live (TTL), and survival status after applying the 10-gene signature to the RNA-seq training dataset.

(B) ROC and AUC based on the 10-gene signature.

(C) KM survival curves for high- and low-risk groups based on the 10-gene signature using the RNA-seq training dataset.

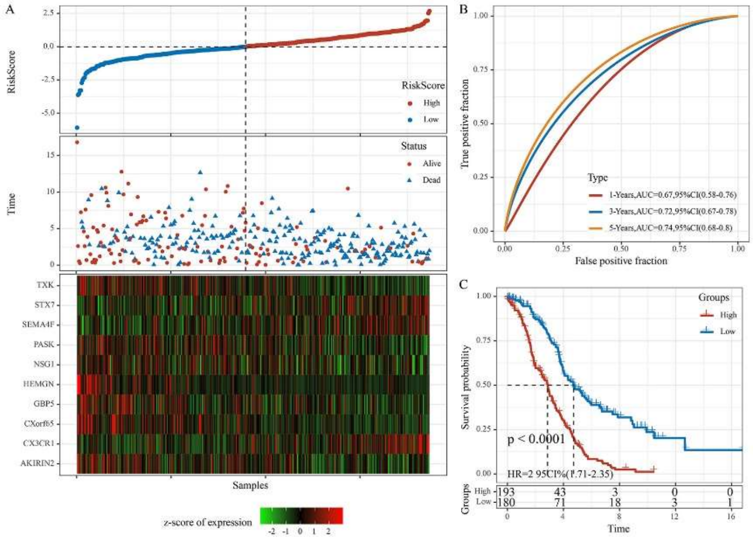

Supplementary Figure S8

(A) RiskScore, TTL, and survival status after applying the 10-gene signature to the RNA-seq validation dataset.

(B) ROC and AUC based on the 10-gene signature.

(C) KM survival curves for high- and low-risk groups based on the 10-gene signature using the RNA-seq validation dataset.

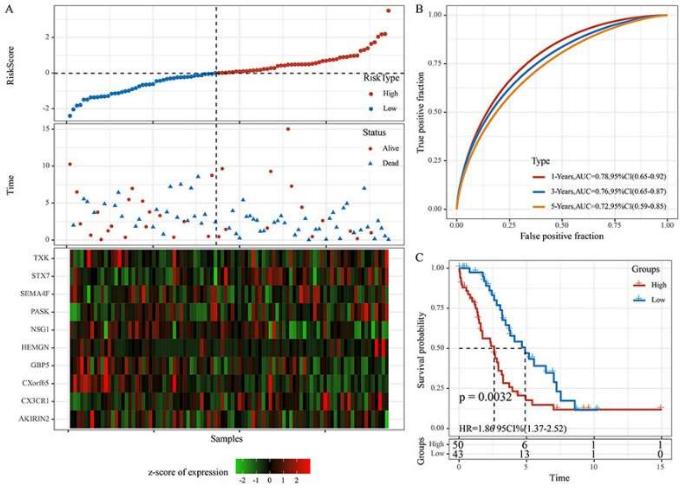

Supplementary Figure S9

(A) RiskScore, TTL, and survival status after applying the 10-gene signature to all the RNA-seq datasets.

(B) ROC and AUC based on the 10-gene signature.

(C) KM survival curves for high- and low-risk groups based on the 10-gene signature using all the RNA-seq datasets.

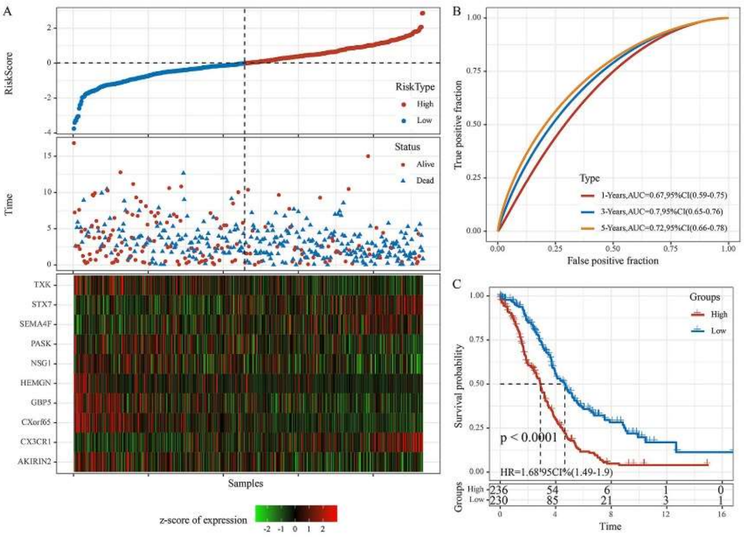

Supplementary Figure S10

(A) RiskScore, TTL, and survival status after applying the 10-gene signature to the independent GSE validation dataset.

(B) ROC and AUC based on the 10-gene signature.

(C) KM survival curves for high- and low-risk groups based on the 10-gene signature using the independent GSE validation dataset.

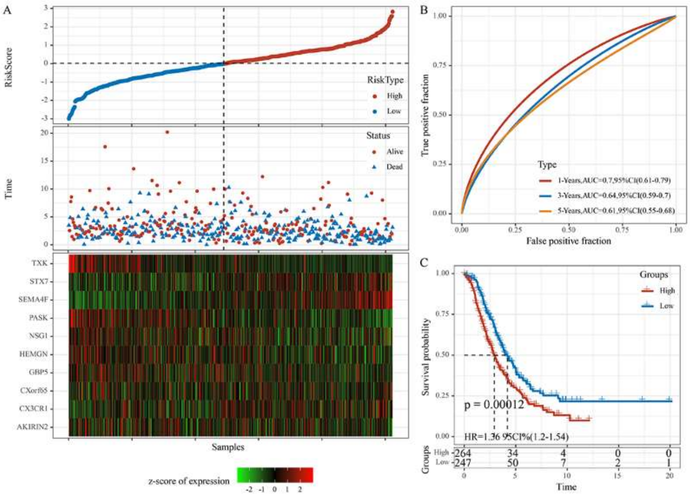

Supplementary Figure S11

KM and ROC curves for RiskScore and the survival of (A) TCGA cohort, (B) ICGC cohort, and (C–F) four GSE cohorts.

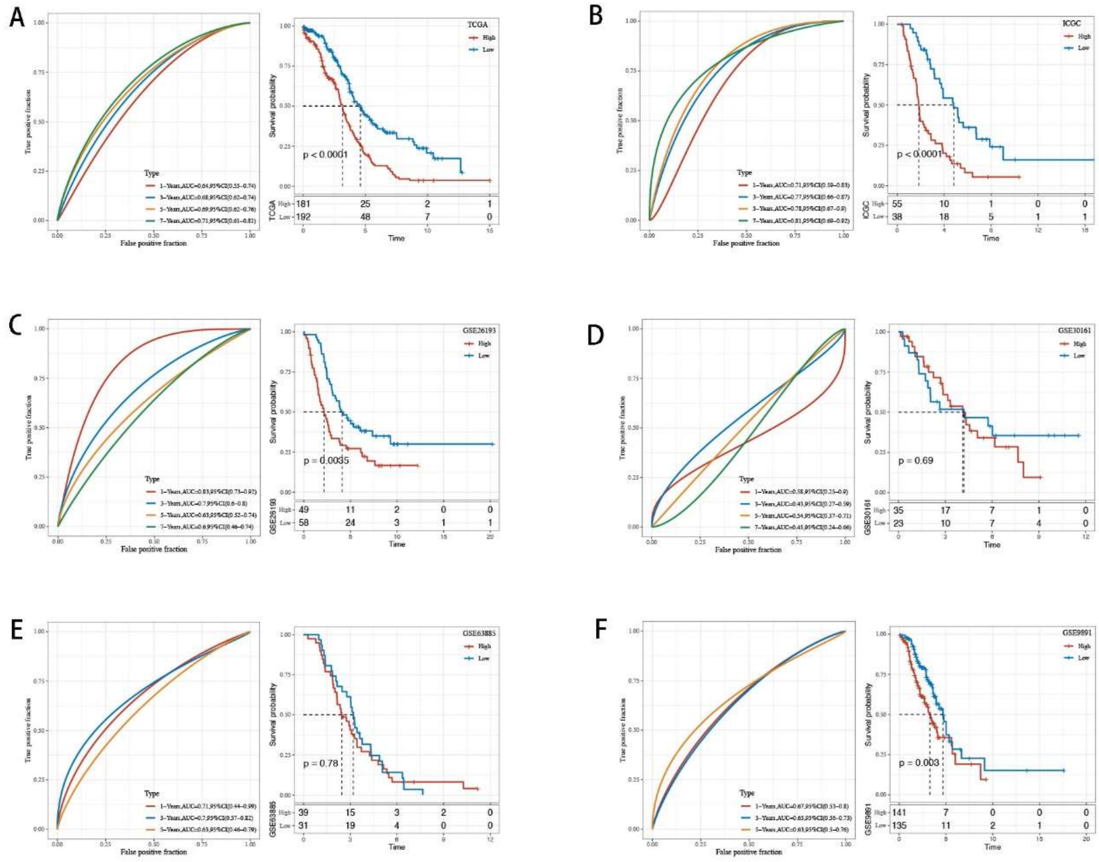

Supplementary Figure S12

Distribution of the RiskScore of the TCGA dataset among clinical features, including (A) grade, (B) stage, (C) ICs, (D) age. Survival analysis of the risk groupings of the TCGA dataset stratified according to clinical characteristics, including (E) age ≤ 60, (F) age > 60, (G) stage I-II, (H) stage III-IV, (I) grade I-II, and (J) grade III-IV.

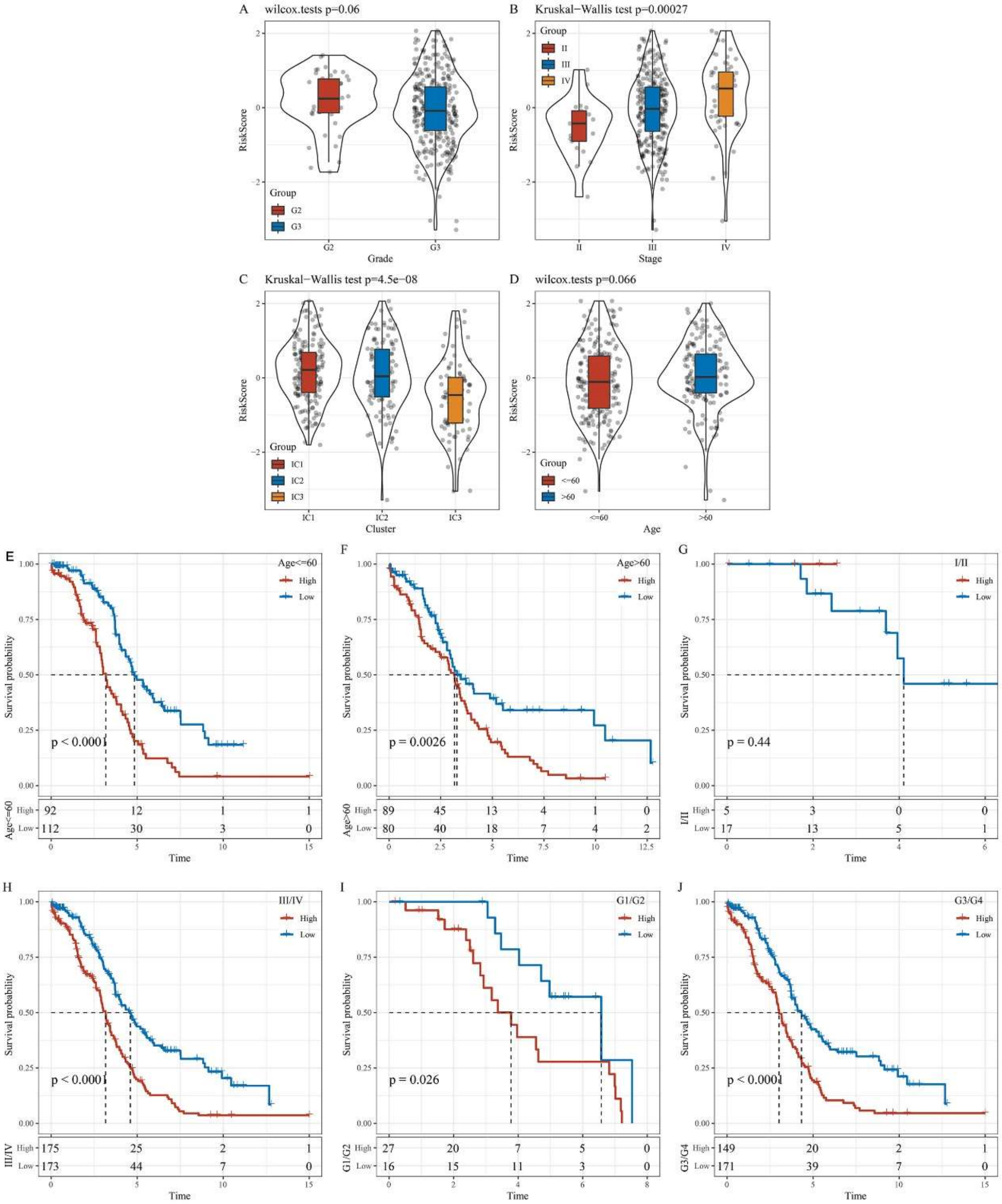

Supplementary Figure S13

Univariate and multivariate analyses of the risk model based on the 10-gene signature using all TCGA datasets.

(A) Univariate Cox regression analysis.

(B) Multivariate Cox regression analysis.

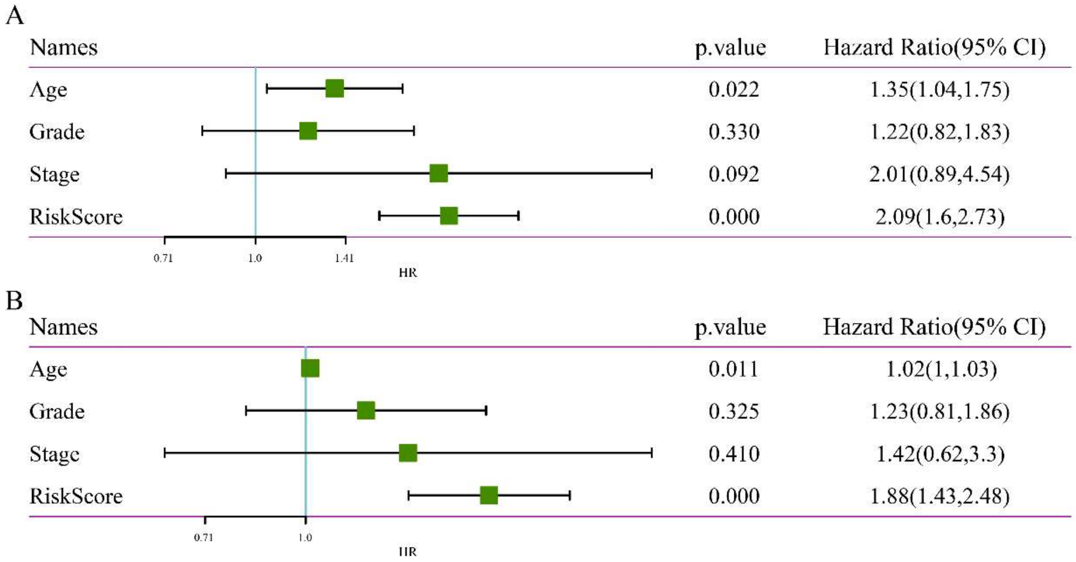

Supplementary Figure S14

Prediction efficacy of the risk model based on the 10-gene signature.

(A) Kaplan–Meier curves of high- and low-risk groups using the Imvigor210 dataset.

(B) Evaluation of the risk model compared with standard prediction models of immunotherapy response using the Imvigor210 dataset.

(C) Corresponding stacked plots of immunotherapy response among high- and low-risk groups in the Imvigor210 dataset.

(D) Correlation between RiskScore, immune score, TMB, and NEO using the Imvigor210 dataset.

CR, complete response; PR, partial response; SD, stable disease; PD, progressive disease.

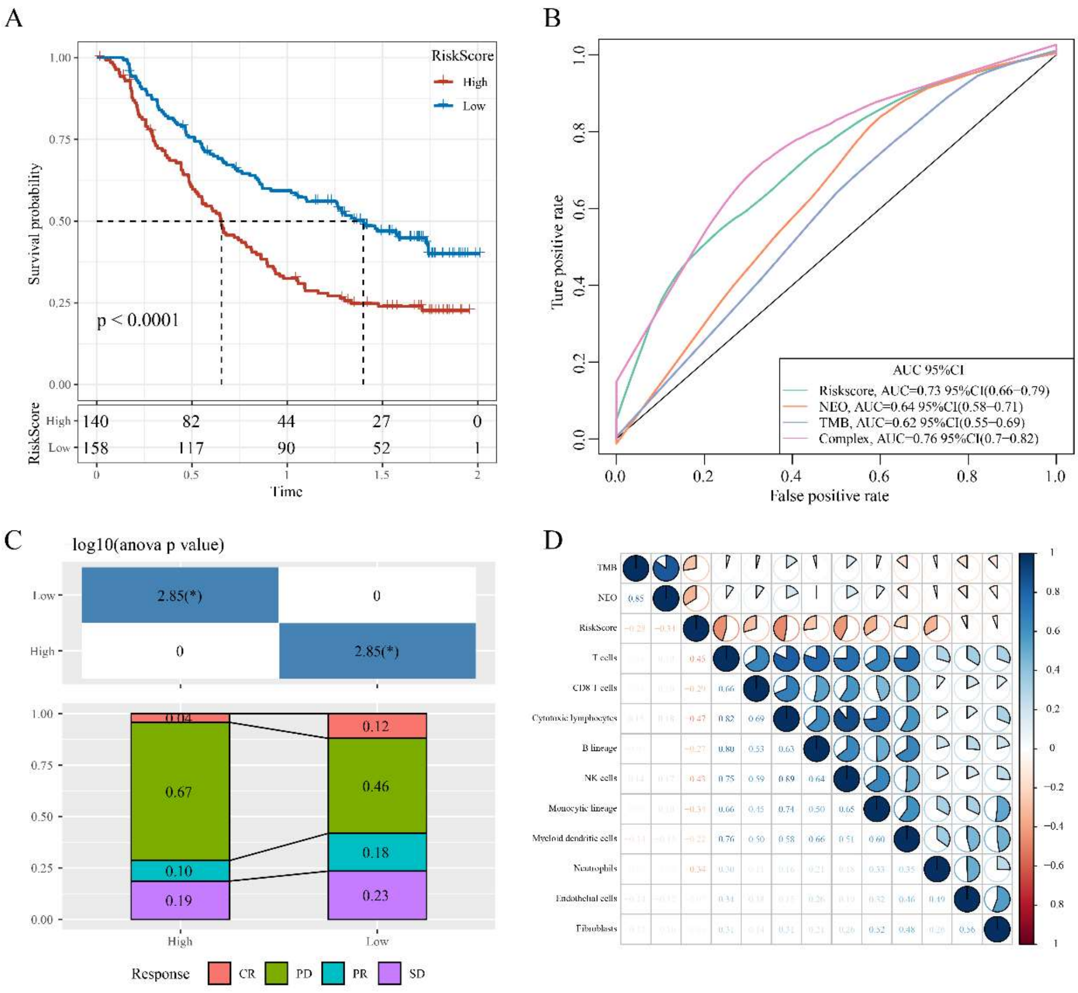

Supplementary Figure S15

Comparison of RiskScore distribution across different subgroups for (A) immunotherapy response, (B) immune cell level, (C) tumour cell level, and (D) immune phenotype.

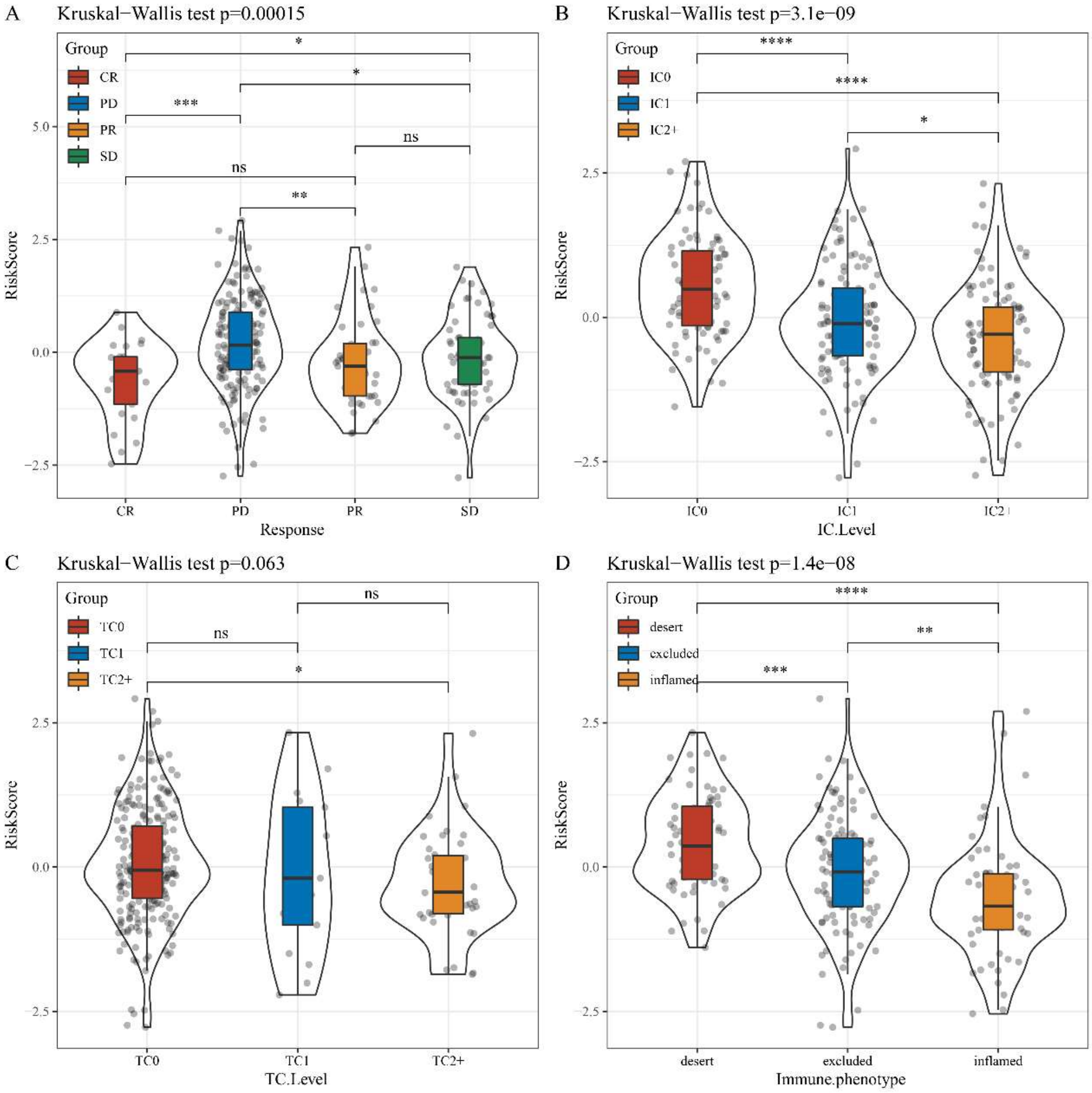

Supplement: Supplementary file 1 [file biomedicines-11-02399-s001.zip › Supplementary Figures.pdf]
