# Supplementary material for: Establishing Molecular Subgroups of CD8+ T Cell-Associated Genes in the Ovarian Cancer Tumour Microenvironment and Predicting the Immunotherapy Response
Source: Biomedicines. 2023 Aug 28;11(9):2399. doi: 10.3390/biomedicines11092399 (PMC10525231; doi:10.3390/biomedicines11092399)
Supplement: Supplementary file 1 [file biomedicines-11-02399-s001.zip › Supplementary Table S1.pdf]

Accounting for CD8+ T cell behaviour in tumour microenvironment helps predicting immunotherapy response in ovarian cancer

Supplementary Table S1. The sequence of primers

TXK-F	GATGGGCAGTTGTTTACCAGCC
TXK-R	ATGGACCACTCCAAACTGACCG
STX7-F	CGAGTAAGAGCCAGTTCCAGAG
STX7-R	TGAATAAGACGGAGGTCATCCTC
SEMA4F-F	GCAGGATGTTGCTGTGCTTCGA
SEMA4F-R	CTGTCCGAATGTCTTGTGGTCG
HEMGN-F	GAACCTGGAGTTTGAGAAACAGAG
HEMGN-R	CCTGTTCTCTGCTGCTTGCGTT
