# Supplementary material for: Establishing Molecular Subgroups of CD8+ T Cell-Associated Genes in the Ovarian Cancer Tumour Microenvironment and Predicting the Immunotherapy Response
Source: Biomedicines. 2023 Aug 28;11(9):2399. doi: 10.3390/biomedicines11092399 (PMC10525231; doi:10.3390/biomedicines11092399)
Supplement: Supplementary file 1 [file biomedicines-11-02399-s001.zip › Supplementary Table S2-3.pdf]

Establishing Molecular Subgroups of CD8+ T Cell-Associated Genes in the Ovarian Cancer Tumour Microenvironment and Predicting the Immunotherapy Response

Supplementary Table S2. Primer sequences for qRT-PCR analysis.

TXK-F	GATGGGCAGTTGTTTACCAGCC
TXK-R	ATGGACCACTCCAAACTGACCG
STX7-F	CGAGTAAGAGCCAGTTCCAGAG
STX7-R	TGAATAAGACGGAGGTCATCCTC
SEMA4F-F	GCAGGATGTTGCTGTGCTTCGA
SEMA4F-R	CTGTCCGAATGTCTTGTGGTCG
HEMGN-F	GAACCTGGAGTTTGAGAAACAGAG
HEMGN-R	CCTGTTCTCTGCTGCTTGCGTT

Supplementary Table S3. Clinical information on 10 patients with OC used for immunohistochemistry

**Clinical characteristics**	**N (%)**
Age	
＜60	7 (70%)
>60	3 (30%)
**Pathological type**	
high grade serous ovarian cancer	10 (100%)
**Clinical Stages**	
**III**	7 (70%)
**IV**	3 (30%)
